# Successful Applications of Food-Assisted and -Simulated Training Model of Thyroid Radiofrequency Ablation

**DOI:** 10.3389/fendo.2022.809835

**Published:** 2022-03-31

**Authors:** Yan-Rong Li, Wei-Yu Chou, Wai-Kin Chan, Kai-Lun Cheng, Jui-Hung Sun, Feng-Hsuan Liu, Szu-Tah Chen, Miaw-Jene Liou

**Affiliations:** ^1^ Division of Endocrinology and Metabolism, Department of Internal Medicine, Linkou Chang Gung Memorial Hospital, Taoyuan, Taiwan; ^2^ College of Medicine, Chang Gung University, Taoyuan, Taiwan; ^3^ Department of Medical Imaging, Chung Shan Medical University Hospital, Taichung, Taiwan; ^4^ School of Medical Imaging and Radiological Sciences, Chung Shan Medical University, Taichung, Taiwan

**Keywords:** thyroid, nodule, radiofrequency ablation, training model, medical education

## Abstract

**Background:**

Radiofrequency ablation (RFA) for benign thyroid nodules is one kind of scarless treatment for symptomatic or cosmetic benign thyroid nodules. However, how to train RFA-naive physicians to become qualified operators for thyroid RFA is an important issue. Our study aimed to introduce a successful training model of thyroid RFA.

**Materials and Methods:**

We used a food-assisted and -simulated training model of thyroid RFA. Chicken hearts were simulated into thyroid nodules, three-layer pork meats were simulated into peri-thyroid structure, and gel bottles were simulated into trachea, respectively. Successful training ablations were defined as chicken hearts that were fully cooked. After repeating training ablations of chicken hearts at least 100 times with the nearly 100% success rates for three young trainees, they served as the first assistant for the real procedures of thyroid RFA and then were qualified to perform thyroid RFA on real patients under the supervision of one experienced interventional radiologist.

**Results:**

23 real patients who received RFA and follow-up at least 6 months after treatment were included in Linkou Chang Gung Memorial Hospital from January 1, 2020 to October 1, 2021. Three young endocrinologists performed thyroid RFA independently. The outcomes were volume reduction rate (VRR), major complications and minor complications. The median VRR at 12 months was 82.00%, two major complications were transient hoarseness, and three minor complications were wound pain. All complications were completely recovered within three days.

**Conclusions:**

For young and RFA-native physicians without any basic skills of echo-guided intervention, this food-assisted and -simulated training model of thyroid RFA was useful for medical training and education.

## Introduction

With the exams by ultrasonography, thyroid nodules are common in general populations, and the prevalence was ever reported 50-60% (1, 2). Among of them, around 7-15% nodules are malignant; therefore, most thyroid nodules are benign (1, 2). For benign thyroid nodules with symptoms and signs of compression or cosmetic problem, treatment should be considered to alleviate the patients’ suffering, such as dyspnea, dysphagia, voice change, or the cosmetic reasons of neck. Radiofrequency ablation (RFA) for benign thyroid nodules is one kind of treatment for symptomatic benign thyroid nodules, especially for cosmetic problem because thyroid RFA is a scarless procedure (3).

Thyroid RFA is a minimally invasive procedure using friction heat productions due to tissue ions agitated by alternating electric current of electrodes and performed with generators and 7-cm electrodes which are 18-gauge, and internally cooled with an active tip measuring 5, 7, or 10 mm (4, 5). The reported volume reduction rate (VRR) by thyroid RFA was from 69% to 78% after 1-year follow-up (6, 7). Some guidelines suggested that thyroid RFA can be used for treating benign thyroid nodules for volume reduction to relieve compression or cosmetic problem (8–10). The core skills are trans-isthmic approach method and moving-shot technique (11). Although knowing these two core techniques, young physicians who have never received step-by-step training by experienced operators and lack in practical experience in real patients may be unable to perform thyroid RFA well and still need a longer learning curve. In addition, young physicians with immature basic and core skills may lead to more complications while learning thyroid RFA in real patients.

As a result, the aim of our study was to investigate the feasibility of the food-assisted and -simulated training model of thyroid RFA in three young endocrinologists who had never performed echo-guided aspiration cytology and thyroid RFA in real patients.

## Materials and Methods

**Figure 1:**
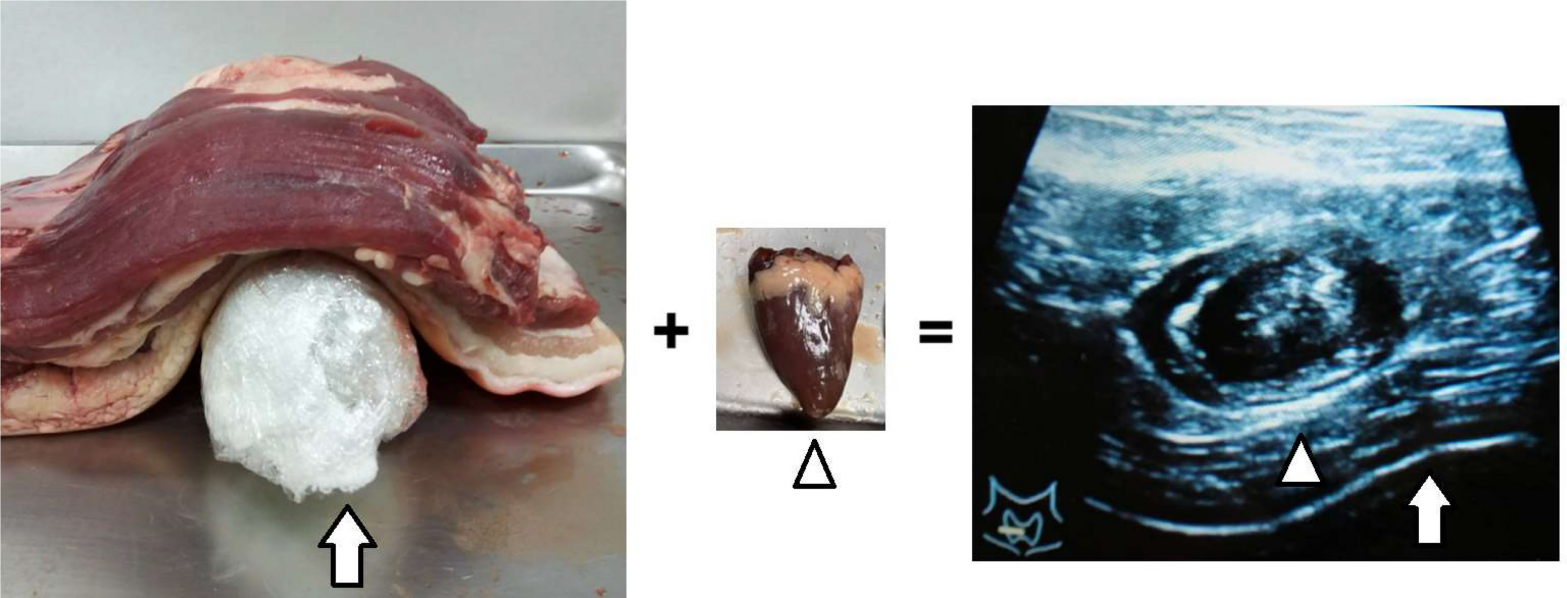
Chicken hearts simulated into thyroid nodules (arrowhead), three-layer pork meats simulated into peri-thyroid skin structure, and gel bottles simulated into trachea (arrow).

**Figure 2:**
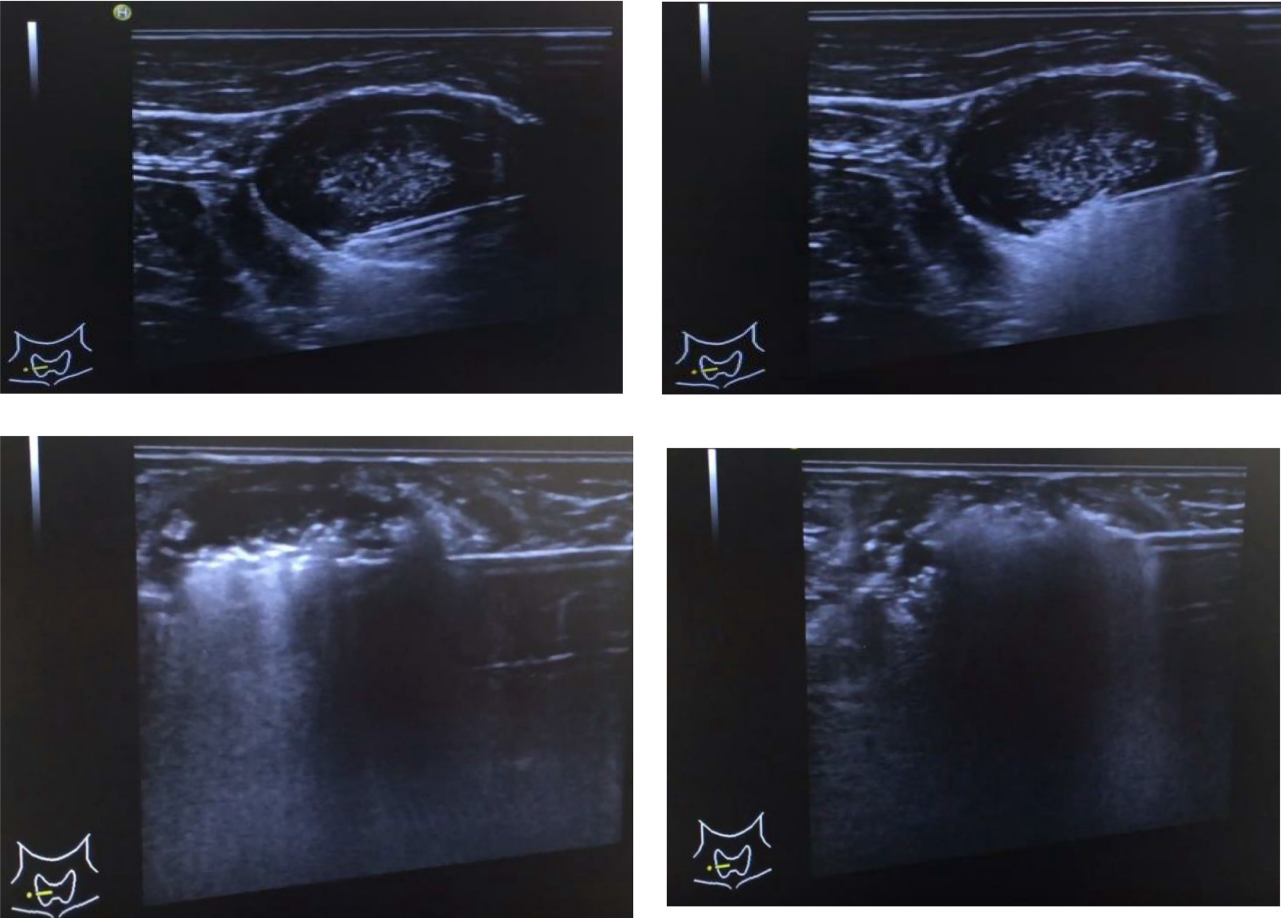
Sequential echo-guided procedures for training the trans-isthmic approach method and the moving shot technique.

**Figure 3:**
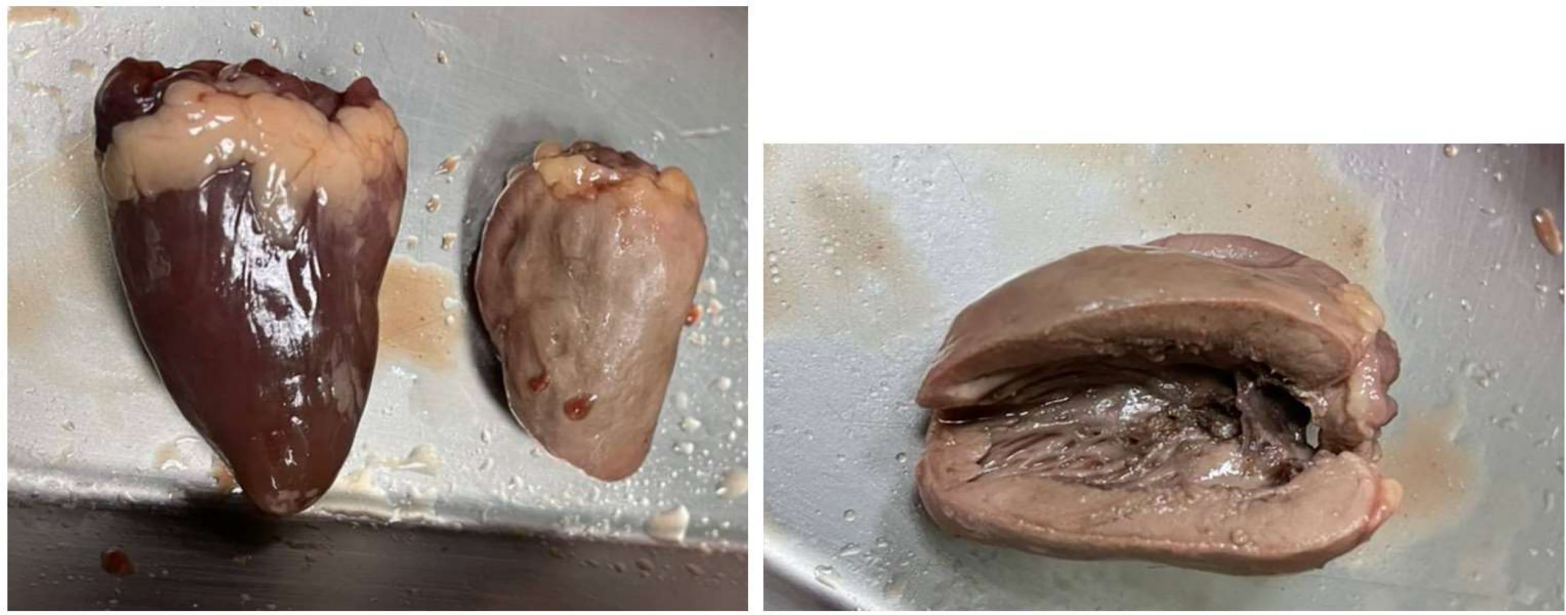
The successful ablations were defined as the color of chicken hearts from bright red to off-white, indicating that the meat was fully cooked.

We used a food-assisted and -simulated training model of thyroid RFA for three young endocrinologists who never performed echo-guided aspiration cytology and RFA. Chicken hearts (size around 2 x 1.5 x 1 cm^3^) were simulated into thyroid nodules and three-layer pork meats (size around 25-30 x 15-20 x 7-10 cm^3^) were simulated into peri-thyroid structure, and gel bottles were simulated into trachea, respectively (**Figure 1**). The generators of RFA were RF Medical Radiofrequency Generator (Mygen M-2004 and M-3004). The electrodes of RFA were RF Electrodes (RFT Series: RFT0707LN). Sequential echo-guided procedures for training the trans-isthmic approach method and the moving shot technique were performed with the food-assisted and -simulated training model of thyroid RFA (**Figure 2**). The successful ablations were defined as the color of chicken hearts from bright red to off-white, indicating that the meat was fully cooked (**Figure 3**).

After repeating ablations of chicken hearts at least 100 times with the nearly 100% success rates for three young endocrinologists and the trainees can performed echo-guided aspirations for cytology of small nodules/lesions on real patients smoothly, they served as the first assistant for the real procedures of thyroid RFA at least five times and then were qualified to perform thyroid RFA on real patients independently under the supervision of one experienced interventional radiologist for patients’ safety. This is a retrospective study of thyroid RFA treatment effectiveness and prognosis after our learning model. Therefore, after training by this model and under the supervision of the leader in our hospital, our hospital permitted that we performed RFA in real patients with patients’ consent and collected data retrospectively after getting certificate of IRB for a retrospective study. This study was approved by the Institutional Review Board, Chang Gung Memorial Hospital, Taiwan (IRB No.: 202101301B0).

Patients with nodular goiter (2 cm in at least one diameter), and predominantly solid nodules which contributed to compressive symptoms and/or cosmetic problem were arranged to receive echo-guided fine-needle aspiration cytology. If the results of cytology showed benign lesions at least twice and patient refused surgical intervention, there were suggested to receive thyroid RFA for the treatment. If patients had multinodular goiter, the predominant and growing nodule was thought to be responsible for symptoms or cosmetic problems and was selected for the thyroid RFA. Patients with nodules > 5 cm in at least one diameter who may need multiple sessions of thyroid RFA were excluded from this study.

From January 1, 2020 to October 1, 2021, a total of 23 real patients who received thyroid RFA and had follow-up for at least 6 months after treatment in Linkou Chang Gung Memorial Hospital were included in the analysis finally.

## Outcome Measurement

The patients were retrospectively reviewed with the medical records to assess the efficacy and the complications of thyroid RFA at the baseline and during the follow-up. The follow-up ultrasonography was performed at 1, 3, 6, and 12 months after thyroid RFA. The volume of nodule and the VRR were calculated using the following equations: volume (mL) = length (cm) × width (cm) × depth (cm) × π/6 and VRR = [(initial volume − final volume)/initial volume] × 100%, respectively.

The major outcomes were changes in the volume of nodules, VRR, major complications, and minor complications one year after thyroid RFA. Major complications included voice change, Horner syndrome, brachial plexus injury, and nodule rupture. Minor complications included wound pain, skin burn and hematoma.

## Statistical Analysis

Categorical data were determined by frequency and percentage. Kolmogorov-Smirnov test was applied to check the normality of continuous data. Continuous data without normal distributions were expressed as medians with interquartile ranges (IQR). Differences in various continuous parameters during the period of follow-up were examined for statistical significance by using Wilcoxon signed rank test as appropriate because of repeated and non-parametric measurements from a single group. A *p* value < 0.05 was considered statistically significant. All data analyses were performed using IBM SPSS Statistics for Windows, Version 22.0. (IBM Corp., Armonk, NY, USA).

## Results

### Baseline Characteristics of Study Patients

23 patients (17 women and 6 men) with 23 benign nodules received thyroid RFA for the treatment. Two trainees performed 8 cases respectively, and the other trainee performed 7 cases. The median age of patients was 51 years (IQR, 43-55 years). The nodules had a median volume of 7.23 mL (IQR, 4.49-13.09 mL) before the thyroid RFA. All nodules were > 50% solidity and 95.65% of nodules were > 90% solidity. All patients had euthyroid status before the treatment of thyroid RFA. The baseline characteristics of study patients are summarized in **Table 1**. No patients had previous surgical intervention, radioactive iodine therapy, thermal/cryo ablation, or radiotherapy which may have an influence on the VRR after thyroid RFA.

**Table 1:** Baseline characteristics in 23 patients with thyroid radiofrequency ablation.

Clinical characteristics	Value
Main nodule for RFA	23
Age at treatment (year)	51 (43-55)
Female gender	17 (73.91)
Largest diameter (cm)	3.50 (2.80-4.01)
Initial volume	7.23 (4.49-13.09)
Small (< 10 ml)	9 (39.13)
Medium (> 10 to ≤ 20 ml)	5 (21.74)
Large (> 20 to ≤ 30 ml)	6 (26.09)
Very large (> 30 ml)	3 (13.04)
Solidity > 50%	23 (100)
Solidity > 90%	22 (95.65)
Euthyroid status	23 (100)

Data were presented as median (interquartile range) or number (percentage).

### Treatment Outcomes and Complications

**Figure 4:**
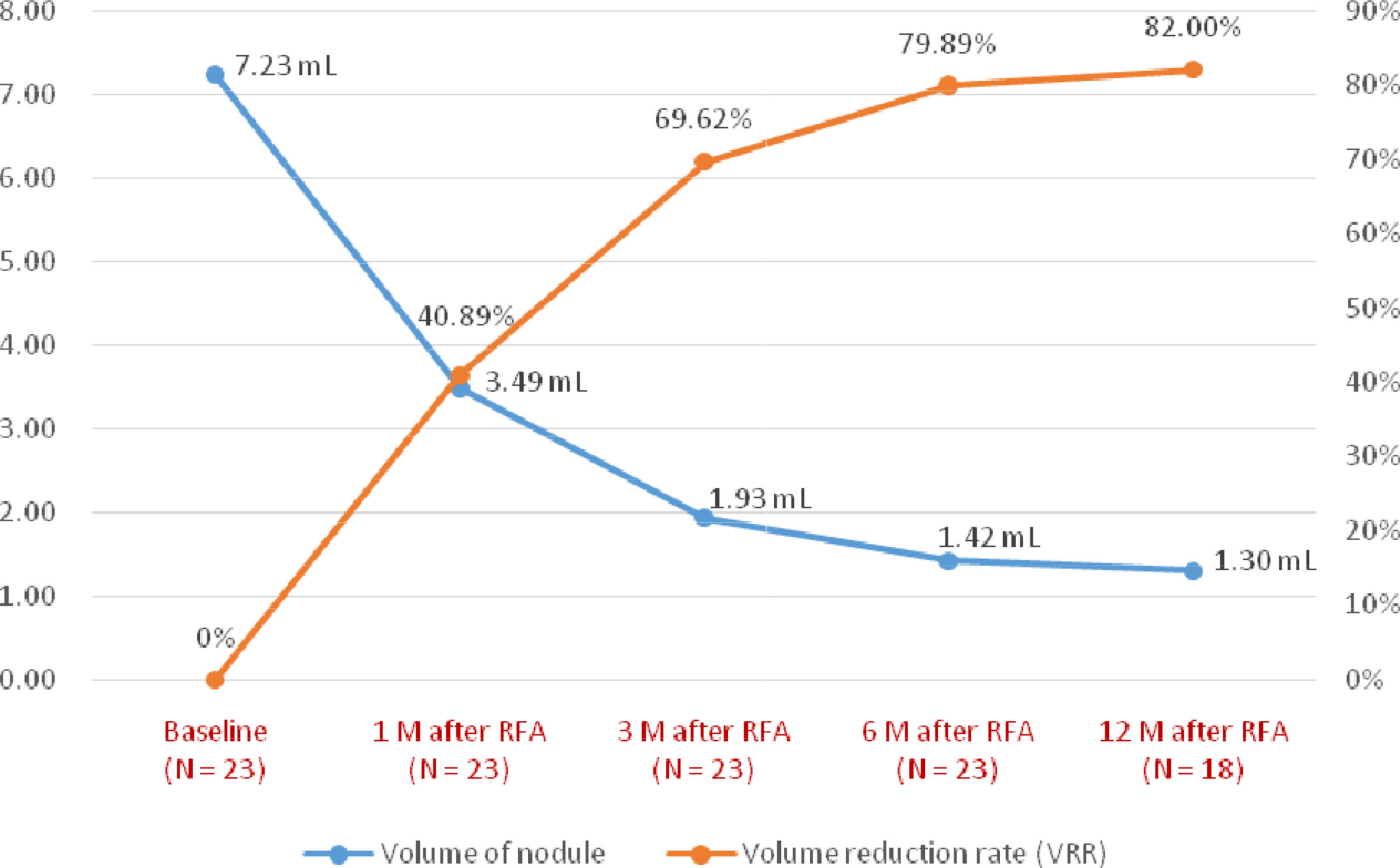
The median changes in the volume of nodules and volume reduction rate during the follow-up.

**Figure 5:**
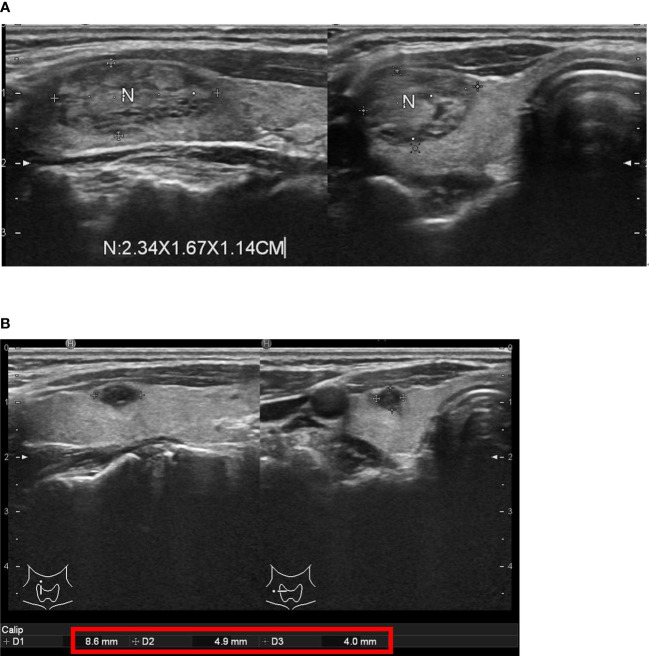
One of our real patients with good response after thyroid radiofrequency ablation (RFA). **(A)** before thyroid RFA **(B)** near 97% volume reduction rate 12 months after thyroid RFA.

The sequential changes in the median volume of nodules before and after thyroid RFA were 7.23 mL (IQR, 4.49-13.09 mL) at baseline, 3.49 mL (IQR, 2.36-6.13 mL) at one month, 1.93 mL (IQR, 1.29-4.45 mL) at three months, 1.42 mL (IQR, 0.63-2.30 mL) at six months and 1.30 mL (IQR, 0.22-2.42 mL) at 12 months, respectively (**Table 2** and **Figure 4**). The sequential median VRR after thyroid RFA were 40.89% (IQR, 32.26-56.78%) at one month, 69.62% (IQR, 60.17-79.06%) at three months, 79.89% (61.91-85.44%) at six months and 82.00% (62.17-87.63%) at 12 months, respectively (**Table 2** and **Figure 4**). Each nodule was ablated with single session. One of our real patients was with good response after thyroid RFA and had near 97% VRR at 12 months (**Figures 5A, B**).

**Table 2:** Outcomes of main nodules after thyroid radiofrequency ablation.

	Initial	1 month after RFA	3 month after RFA	6 month after RFA	12 month after RFA
	(N = 23)	(N = 23)	(N = 23)	(N = 23)	(N = 18)
Volume of nodule (mL)	7.23 (4.49-13.09)	3.49 (2.36-6.13)*	1.93 (1.29-4.45)*	1.42 (0.63-2.30)*	1.30 (0.22-2.42)*
VRR (%)	0	40.89 (32.26-56.78)*	69.62 (60.17-79.06)*	79.89 (61.91-85.44)*	82.00 (62.17-87.63)*
Major complications					
Voice change	2	0	0	0	0
Horner syndrome	0	0	0	0	0
Brachial plexus injury	0	0	0	0	0
Nodule rupture	0	0	0	0	0
Minor complications					
Wound pain	3	0	0	0	0
Skin burn	0	0	0	0	0
Hematoma	0	0	0	0	0
Euthyroid status (%)	100	100	100	100	100

RFA, radiofrequency ablation; VRR, volume reduction rate; N, number.

Data were presented as median (interquartile range), number, or percentage.

*p value < 0.05 when compared with the initial nodules.

Hoarseness could be one kind of major complications after thyroid RFA. There were two patients having transient voice change and both patients recovered totally; one was within five minutes and another was within one day. There were three patients having wound pain and needed non-steroidal anti-inflammatory drugs (NSAIDs) for pain control. Wound pain of these three patients was improved and all patients discontinued NSAIDs within three days. During the follow-up, there was no event of Horner syndrome, brachial plexus injury, nodule rupture, skin burn or hematoma. Due to the small case number of each trainee, the differences of efficacy/complications among these three physicians were not statistically significant.

## Discussion

There could be a long learning curve for some physicians to perform thyroid RFA. One recent study showed that there was a measurable learning curve in thyroid RFA until a total of 90 patients for the efficacy of benign thyroid nodules (12). In other words, there could be a minimum number of thyroid RFA for a physician to optimize patients’ outcomes. The acceptable VRR after thyroid RFA is at least ≥ 50% at 12 months (13). This concept is like a previous study for evaluation of outcomes in thyroidectomy and the result showed that a surgeon volume threshold (> 25 total thyroidectomies per year) is correlated with improved patients’ outcomes (14).

The strength of our study is that it is the first established food-assisted and -simulated training model of thyroid RFA and the result showed successful outcomes (the median VRR at 12 months: 82.00%) for medical training and education. This kind of simulated training model may shorten the learning curve in real patients and could prevent the bad outcomes in the initial 90 patients undergoing thyroid RFA. In other words, by our learning model, trainees can improve their major skills and ability and may be able to prevent excessive trial-and-error learning in the initial 90 real patients.

In Taiwan, thyroid RFA developed later than Korea. In the search of literatures, the first documented case of thyroid RFA in Taiwan was published in 2016 (15). In Korea, Professor Jung Hwan Baek started thyroid RFA since 2002 (16) and ever published a large population study of thyroid RFA in 2008 (17). Nowadays, many guidelines in different countries have suggested thyroid RFA can be a standard of care to treat benign thyroid nodules for volume reduction to relieve compression or cosmetic problem (3, 8–10). However, in Taiwan, for most clinical physicians who have interests in thyroid RFA, they still have no idea how to learn thyroid RFA efficiently. Traditionally, learning a new invasive procedure requires someone who has a lot of experience to teach the trainee step by step. Two most famous physicians in Taiwan, both Dr. Wei-Che Lin and Dr. Kai-Lun Cheng have performed over one hundred cases of thyroid RFA and mainly contributed to publish a multicenter study of 762 cases after thyroid RFA which established the efficacy and safety of RFA for benign thyroid nodules in Taiwan (18) but they can not teach every trainee by the real patients in Taiwan step by step. Therefore, under the supervision of Dr. Kai-Lun Cheng, one experienced interventional radiologist in Taiwan, we worked together to build this kind of food-assisted and -simulated model for thyroid RFA training. Setting a good training model to learn a new invasive procedure is very important. A good simulated training model consists of hands-on training, deliberate practice, training to proficiency, cognitive teaching, and effective provision of feedback (19). Our training model meets the above points and showed good clinical results after training. The key components of our training model were as the followings: ablations of chicken hearts at least 100 times with the nearly 100% success rates, already performing trans-isthmic approach method with moving-shot technique very well, and understanding the critical structures associated with thyroid RFA, such as danger triangle, middle cervical sympathetic ganglion, and vagal nerve after serving as the first assistant for the real procedures of thyroid RFA at least five times. Besides, this training model is not expensive, easy to prepare materials and to facilitate the promotion of education. Each round of practice cost around 2500-3500 New Taiwan Dollar and can train multiple trainees at the same time and in the same place. Using this model, we successfully trained and cultivated three physicians simultaneously.

This present study had several major limitations. First, although we tried our best to simulate the peri-thyroid structure but we were unable to simulate the blood vessel, nerves, and esophagus. Therefore, the trainees needed to imagine the locations of the common coratid artery, internal jugular vein, vagal nerve, cervical sympathetic ganglion, danger triangle, and esophagus while practicing the trans-isthmic approach method and moving-shot technique. Second, although the current results of our study showed good applications of our training model with the median VRR 82.00% in real patients, the number of our study subjects is only 23 patients. Third, the longest time of follow-up in our study was 12 months which means we could not confidently evaluate whether the nodular regrowth will occur. Despite these disadvantages, our present study can still provide a valuable training model for those who want to learn how to practice thyroid RFA but do not know where to start.

## Conclusions

For young and RFA-native physicians without any basic skills of echo-guided intervention, this food-assisted and -simulated training model of thyroid RFA was successful for medical training and education.

## Data Availability Statement

The raw data supporting the conclusions of this article will be made available by the authors, without undue reservation.

## Ethics Statement

This study was approved by the Institutional Review Board, Chang Gung Memorial Hospital, Taiwan (IRB No.: 202101301B0). The patients/participants provided their written informed consent to participate in this study.

## Author Contributions

The conceptualization and design of the study was headed by Y-RL, W-KC, K-LC, and M-JL. The manuscript draft was written by Y-RL and M-JL. While the data analysis and interpretation were done by Y-RL, W-YC, W-KC, K-LC, J-HS, F-HL, S-TC, and M-JL. All authors contributed to the article and approved the submitted version.

## Conflict of Interest

The authors declare that the research was conducted in the absence of any commercial or financial relationships that could be construed as a potential conflict of interest.

## Publisher’s Note

All claims expressed in this article are solely those of the authors and do not necessarily represent those of their affiliated organizations, or those of the publisher, the editors and the reviewers. Any product that may be evaluated in this article, or claim that may be made by its manufacturer, is not guaranteed or endorsed by the publisher.
